# Practical guidance for the implementation of stress echocardiography

**DOI:** 10.1007/s12574-018-0382-8

**Published:** 2018-06-06

**Authors:** Kengo Suzuki, Yutaka Hirano, Hirotsugu Yamada, Mitsushige Murata, Masao Daimon, Masaaki Takeuchi, Yoshihiro Seo, Chisato Izumi, Makoto Akaishi

**Affiliations:** 1 Division of Cardiology, St. Marianna University School of Medicine, Kawasaki, Japan; 2 Division of Cardiology, Department of Medicine, Faculty of Medicine, Kindai University, Osakasayama, Japan; 3 Department of Cardiology, Tokushima University Hospital, Tokushima, Japan; 4 Department of Laboratory Medicine, School of Medicine, Keio University, Tokyo, Japan; 5 Department of Clinical Laboratory, Faculty of Medicine, The University of Tokyo, Tokyo, Japan; 6 Department of Laboratory and Transfusion Medicine, Hospital of the University of Occupational and Environmental Health, Kitakyushu, Japan; 7 Cardiovascular Division, Faculty of Medicine, University of Tsukuba, Tsukuba, Japan; 8 National Cerebral and Cardiovascular Center, Suita, Japan; 9 Tokai University Tokyo Hospital, 1-2-5 Yoyogi Shibuya-ku, Tokyo, Japan

**Keywords:** Peak oxygen uptake, METS (metabolic equivalents), Ischemic heart disease, Valvular heart disease, Cardiomyopathy, Dobutamine, Stress echocardiography

## Abstract

Exercise stress testing has been widely undertaken for the diagnosis of heart diseases. The accurate assessment of clinical conditions can be conducted by comparing the findings obtained from the results of stress echocardiography with the changes in the blood/heart rate and electrocardiograms. Numerous overseas studies have reported the utility of stress echocardiography in diagnosing myocardial ischemia; in Japan, the use of this modality for this purpose was included in the national health insurance reimbursable list in 2012. Nevertheless, stress echocardiography is far from being a widespread practice in Japan. This might be due to insufficient equipment (e.g., ergometers, space for test implementation) at each medical institution, shortage of technicians and sonographers who are well experienced and who are responsible for obtaining images during stress testing. The other possible reasons include the limited evidence available in Japan and the lack of a standardized testing protocol. Further dissemination of the practice of exercise stress echocardiography in this country is deemed necessary to establish satisfactory evidence for the use of stress echocardiography in the Japanese population. To this end, efforts are underway to develop a standardized protocol and report format to be adopted throughout Japan. We here present a guideline created by the Guideline Development Committee of the Japanese Society of Echocardiography that describes safe and effective stress echocardiography protocols and report formats. The readers are encouraged to perform exercise stress echocardiography using the proposed template for consensus document and report attached to this guideline.

## Introduction

During exercise to sustain the increased metabolic demand of the tissues, increased oxygen and nutrient delivery are accomplished by increasing cardiac output (CO) and blood flow to the microvascular surface area, in addition to increased O_2_ extraction in the active muscles (heart, respiratory, abdominal and skeletal muscles).

Furthermore exercise-associated tachycardia and blood pressure elevation lead to valvular regurgitation and increased myocardial oxygen consumption. Examining cardiac function during exercise as well as at rest is thus important for selecting suitable therapy for heart disease. Exercise stress testing has been widely undertaken for the diagnosis of heart diseases [1, 2]. In the circumstances where exercise stress cannot be performed, detailed evaluations under drug stimulation have been carried out to make an assessment of cardiac reserve. The accurate assessment of clinical conditions can be conducted by comparing the findings obtained from the results of stress echocardiography with the changes in the blood/heart rate and electrocardiograms (ECGs). Numerous overseas studies have reported the utility of stress echocardiography in diagnosing myocardial ischemia; [3] in Japan, and the use of this modality for this purpose was included in the national health insurance reimbursable list in 2012.

Nevertheless, stress echocardiography is far from being a widespread practice in Japan. This might be due to insufficient equipment (e.g., ergometers, space for test implementation) at each medical institution, shortage of technicians and sonographers who are well experienced and who are responsible for obtaining images during stress testing. The other possible reasons include the limited evidence available in Japan and the lack of a standardized testing protocol. Further dissemination of the practice of exercise test echocardiography in this country is deemed necessary to establish satisfactory evidence for the use of stress echocardiography in the Japanese population. To this end, efforts are underway to develop a standardized protocol and report format to be adopted throughout Japan. We here present a guideline created by the Guideline Development Committee of the Japanese Society of Echocardiography that describes safe and effective stress echocardiography protocols and report formats.

## Overview

### Stressing methods for stress echocardiography

Stress echocardiography is mainly conducted during exercise or under drug stimulation. For the pharmacological stress testing, adenosine was once administered. At present, dobutamine is the only drug used for this purpose (dobutamine stress testing is described below for reference purposes). The other stressing methods include pacing, hyperventilation, and the use of a cold pressor (Table 1); they are employed only under special circumstances.

**Table 1:** Stressing methods for stress echocardiography

	Exercise	Pharmacological	Others
Stressing methods	Treadmill	Dobutamine	Pacing
	Ergometer	Dipyridamole	Hyperventilation/cold pressor
	Handgrip	Adenosine	Leg raising
Type of stress	Physiological	Nonphysiological	Nonphysiological
Heart rates	Increased	Increased	Vary depending on stress
Blood pressures	Increased	Not necessarily increased	Vary depending on stress
Image quality	Can be unassessable	Adequate for assessment in many cases	Adequate for assessment in many cases
Venous route	Not needed	Needed	Needed/not needed
Other	In patients capable of performing exercise, objective exercise tolerance and relationship to symptoms can be assessed	Cardiac stress can be imposed in the patients incapable of performing exercise	Leg raising is a simple technique to increase preload

### Exercise stress testing methods

Exercise stress testing can be performed with isometric (e.g., handgrip) or isotonic (e.g., treadmill, ergometer) exercise. To evaluate the cardiac pumping capacity, we need to increase CO by raising oxygen consumption in the peripheral tissues. When we intend to induce myocardial ischemia by elevating myocardial oxygen consumption, myocardial oxygen demand needs to be raised. Myocardial oxygen consumption largely depends on left ventricular (LV) wall stress and heart rate rather than CO; therefore, to evoke myocardial ischemia, it is necessary to increase LV wall stress and heart rate, i.e., to achieve higher blood pressure and heart rate via exercise. While the cardiac pumping capacity is assessed, it is essential to raise CO.

Generally, an isometric exercise is characterized by smaller increases in heart rate, compared with the magnitude of increases in systolic blood pressure, at higher exercise intensity. In an isotonic exercise, greater exercise intensity is associated with greater CO; both blood pressure and heart rate typically show a linear increase in proportion to exercise intensity. In principle, venous return is increased, resulting chiefly in ventricular expansion. This venous return increase is more conspicuous when the subject is in a recumbent position than on standing.

Isometric exercise stress, which is associated with greater blood pressure elevation than with isotonic stress, may allow improved detection rates for wall motion abnormalities since such abnormalities are more readily visualized in the presence of elevated LV wall stress, as well as ischemia, which can be induced with isometric exercise by increasing myocardial oxygen demand.

To evaluate pumping ability of the heart, an increase in stroke volume (SV) should be monitored. In the exercise stress testing for aortic stenosis (AS), elevation in pressure gradient and change in valve area resulting from increased SV are examined; therefore, the type of stress that raises SV (e.g., stress on recumbent exercise) is more desirable than the type that merely raises heart rate (e.g., pacing).

On the other hand, in the stress testing for mitral stenosis (MS), where the diastolic period is markedly shortened with increased heart rate, the magnitude of the effect of shortened diastolic period exceeds that of stress from CO increase, resulting in a greater left atrioventricular pressure gradient. In other words, the stress testing for MS is for the assessment of cardiac capacity under the conditions of elevated heart rate.

Mitral regurgitation (MR) can have an organic or functional cause, and how imposed stress will interact with such causes is unpredictable. In this context, it may be best to apply types of stress closest to those encountered in daily activities (e.g., stress that raises cardiac output) in evaluating patients’ symptoms.

In many cases, stress testing for myocardial disease is conducted to assess the contractile reserve of the myocardium itself. Pharmacological stress testing with low-dose catecholamines is therefore desirable.

### Physiology of exercise stress

During exercise, depending on its intensity, the volume of oxygen uptake (*V*O_2_) increases. The *V*O_2_ at the time when the target heart rate is reached in symptom-limited stress testing is called the peak *V*O_2_. The highest *V*O_2_ achieved with exercise of increasing intensity (plateau *V*O_2_) is called the maximal *V*O_2_.

Peak *V*O_2_ is determined by the maximum oxygen transport capability and the maximum oxygen utilization capability during exercise. The maximum oxygen transport capability depends on the vasodilatory ability and the ability to efficiently perfuse the skeletal muscles with blood, in addition to CO reserve. The maximum oxygen utilization capability relies on the ability of the active muscles to take up oxygen effectively. Possible mechanisms for a decrease in peak *V*O_2_ in patients with heart failure thus include: lower CO, lower blood pressure, reduced vasodilation due to endothelial dysfunction, decreased muscle volume due to restriction of physical activity and disuse atrophy, skeletal mitochondrial change due to chronic hypoperfusion, and decreased activity of enzymes, such as the enzymes of oxidative phosphorylation, which are involved in muscular energy metabolism.

Stress testing should be performed using a method suitable for the purpose of testing, selected based on predicted changes in variables in response to a given stress. In selecting the testing method, it is imperative to give top priority to the safety of the patient, taking into consideration his/her condition. For the elderly, cycle ergometer exercise is recommended as falls are unlikely, rather than a treadmill exercise, which in the elderly is associated with a risk of falls and possibly excessive workload in terms of oxygen consumption.

Ergometer and treadmill exercise tests achieve similar heart rate and ventilation at the highest exercise intensity, but the peak *V*O_2_ at that point is generally 5–11% higher with treadmill exercise [4]. In other words, the peak *V*O_2_ determined using ergometer exercise is lower than that obtained from the results of a treadmill. These two stressing methods also differ in terms of the effect of the subject’s body weight on *V*O_2_ per unit weight. With treadmill exercise, workload is produced by walking; that is, the patient does work by shifting his/her weight. The workload is thus expressed by ‘work rate’, i.e., body weight × walking velocity. The heavier the subject, the greater is the workload. When the incline and walking speed are changed, the *V*O_2_ per weight (METS; metabolic equivalents) changes, while the relationship between the workload and *V*O_2_ remains the same regardless of subject’s body weight. Meanwhile, using cycle ergometer exercise, where the subject pedals in a sitting position, the relationship between the workload and *V*O_2_ per body weight varies depending on the body weight: *V*O_2_ per weight becomes smaller at greater weights [5].

When treadmill exercise is used for echocardiography, the patient moves to the testing bed and receives echocardiography after finishing the exercise; the data obtained are thus post-exercise echocardiograms. With supine ergometer exercise, stress testing echocardiograms can be acquired during exercise, a distinct feature of this test.

### Objectives of exercise stress echocardiography

#### Ischemic heart disease^1^

1. The diagnosis of coronary artery disease
  Patients in whom echocardiographic assessment is less reliable when detecting ischemia: in those administered with digoxin, those with pre-excitation (Wolff–Parkinson–White) syndrome and those with complete left bundle-branch block.
  The meta-analysis results are available for diagnostic accuracy of exercise echocardiography for identifying coronary artery disease [7–9] The sensitivity and specificity were reported to be 87 and 72%, respectively, in the meta-analysis by de Jong et al. [8] and 82.7 and 84.0%, respectively, by Heijenbrok-Kal et al. [9]. Table 2 summarizes the findings on the diagnostic accuracy of different stress echocardiographic modalities [9].

**Table 2:** Diagnostic accuracy of various stress echocardiographic modalities

	Number of studies	Sensitivity (%) (95% confidence interval)	Specificity (%) (95% confidence interval)
Exercise stress	55	82.7 (80.2–85.2)	84.0 (80.4–87.6)
Adenosine	11	79.2 (72.1–86.3)	91.5 (87.3–95.7)
Dipyridamole	58	71.9 (68.6–75.2)	94.6 (92.9–96.3)
Dobutamine	102	81.0 (79.1–82.9)	84.1 (82.0–86.1)
2. Prognosis and risk assessment.
  In several studies published to date on the use of exercise stress echocardiography for the assessment of prognosis and risk, this modality has been reported to be useful in assessing numerous diseases. Negative results on exercise stress echocardiography predict favorable patient outcomes [10]. Exercise stress testing has also been found to be of prognostic value for women; [11] the elderly; [12] patients with diabetes mellitus [13], angina pectoris [14], or acute myocardial infarction; [15–17] and in patients who have undergone coronary artery bypass grafting (CABG) [18]. Monitoring such subpopulation for abnormal wall motion with exercise stress echocardiography is useful for predicting cardiovascular events.
3. Preoperative evaluation for cardiovascular surgery and other major surgery.
  Stress echocardiography has been reported to be useful for preoperative risk assessment prior to cardiovascular surgery [19, 20] and other major surgery [21, 22].
4. Assessment of residual myocardial ischemia after a revascularization procedure such as percutaneous coronary intervention and CABG.
  Exercise stress echocardiography has been reported to be useful in evaluating residual myocardial ischemia in patients following revascularization such as percutaneous coronary intervention [23] and CABG [24].
5. Diagnosis of the location of myocardial ischemia.
  In identifying the location of myocardial ischemia, the utility of exercise stress echocardiography and coronary arteriography has been compared, and it has been concluded that the former is very useful, with a high consistency shown between myocardial ischemia location and coronary lesion [25].
6. Assessment of myocardial viability.
  Regarding myocardial viability assessment, several studies found that low-dose dobutamine stress testing was useful [26]. Some studies reported that myocardial viability can be evaluated based on wall motion assessment performed during low-level ergometer exercise [27, 28].
7. Relationship with chest symptoms on exertion.
  Exercise echocardiography is useful for the diagnosis of patients with dyspnea on exertion and can predict outcomes of patients with dyspnea [29].

#### Cardiomyopathy

1. *Dilated cardiomyopathy* Evaluation of contractile reserve, exercise-induced dyssynchrony, MR, and pulmonary hypertension (PH).^2^
2. *Hypertrophic cardiomyopathy* Evaluation of exercise-induced LV outflow tract (LVOT) obstruction, MR, PH,^2^ and regional wall motion.
3. Relationship with symptoms on exertion.

#### Valvular heart disease

1. *MR* Assessment of exercise-induced changes in MR and PH.^2^ Prediction of postoperative LV function.
2. *MS* Assessment of left atrioventricular pressure gradient in patients who show findings on resting echocardiograms that are disproportionate to symptoms; and assessment of PH.
3. *AS* Risk assessment for asymptomatic, severe AS.
4. *Aortic regurgitation (AR)* Assessment of contractile reserve and prediction of decline in cardiac function after aortic valve replacement.
5. Relationship to symptoms on exertion.

#### Pulmonary hypertension

1. *PH* Diagnosis of exercise-induced PH,^2^ prediction of prognosis, and assessment of treatment effect.
2. Relationship to symptoms on exertion.

### Ensuring safety in exercise stress echocardiography

**Fig. 1:**
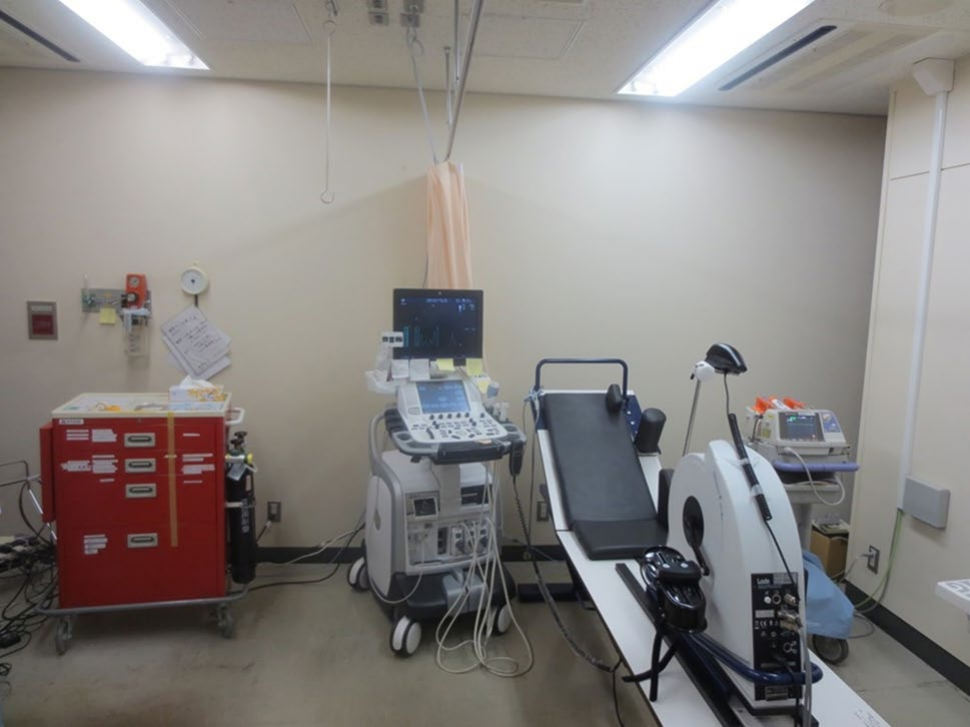
Stress Echo Room (US Center, St. Marianna University School of Medicine). Exercise stress testing should be performed in a sufficiently spacious room with the following equipment readily available: an emergency cart with emergency drugs and airway management devices, exercise stress monitoring system (automated sphygmomanometer, 12-lead ECG monitor), defibrillator, and oxygen tanks

The safety of exercise stress echocardiography has been established. The incidence rate of serious complications (e.g., serious arrhythmias, myocardial infarction) has been reported to be 0.04% during stress exercise testing, 0.01% after stress exercise testing, and ≤ 0.2% for overall complications [7]. Particular attention should be paid to ensure the safety in patients who are unable to perform exercise stress testing for physical reasons, since they are at higher risk of developing serious complications. For such patients, we recommend that the testing is performed in a sufficiently spacious room with the following equipment readily available: an emergency cart with emergency drugs and airway management devices, exercise stress monitoring system (automated sphygmomanometer, 12-lead ECG monitor), defibrillator, and oxygen tanks (Fig. 1).

### Contraindications for exercise stress echocardiography

To perform exercise stress testing safely and acquire the data useful for diagnosis, it is important to predetermine if the patient is eligible for stress testing. Patients with the conditions listed below are at high risk of stress-induced cardiac events; in such patients stress testing is contraindicated as a rule.

1. Acute coronary syndrome within 48 h of onset.
2. Poorly controlled heart failure/dyspnea.
3. Poorly controlled hypertension.
4. Symptomatic severe AS.^3^
5. Severe obstructive hypertrophic cardiomyopathy (LVOT pressure gradient > 90 mmHg).
6. Life-threatening arrhythmias.
7. Acute phase of acute aortic dissection, aortic aneurysm with threatened rupture.
8. Inability to exercise.^4^
9. Without prior written consent to undergo the exercise stress testing.
10. Any other patients who are identified as ineligible by their attending physicians.

## Implementation of exercise stress testing

### Before exercise stress testing

1. General precautions.
  Before stress testing, the clinical diagnosis, purpose of the test, medical history, and the patient’s current medications should be confirmed. Prior written consent must be obtained after an explanation of the specific stress testing method and possible side effects should be fully explained. Prior to implementation of exercise stress testing, the physician in charge must ask the patient’s clinical course including recent chest symptoms and the frequency of such symptoms. As a rule, a symptom-limited exercise stress test (e.g., leg exhaustion, dyspnea, chest pain) should be performed. The patients are advised to wear comfortable pants and a patient's gown for quick recording of echocardiograms.
2. Measurements of vital signs.
  If the patient suffers from tachycardia or presents blood pressure that differs from his/her normal level, its cause should be investigated and a decision should be made whether exercise stress testing may or may not be performed.
3. To make it clear whether the patients correctly understood the purpose of testing and procedure.
  It is indispensable to provide special care for patients undergoing the testing to relieve their anxieties.
4. To confirm the accuracy of echocardiographic images.
  You should confirm the chest areas where the transducer is to be properly placed to obtain ultrasound views and check that the chest ECG electrodes will not interfere with the manipulation of the transducer.

### Stress testing procedures

#### Treadmill exercise stress

1. Instruct the patient to stand on the belt and then commence the stress test.
2. Increase the workload in a stepwise manner according to the Bruce protocol or another appropriate protocol. The Bruce protocol, which is originally intended to induce ischemia in patients with heart disease, gradually increases the workload in large increments. It should be noted that in the first step, the patient is required to walk at a rate of 1.7 miles/h (2.7 km/h) at a 10% incline, which is equivalent to a workload of approximately 17–18 mL/min/kg body weight of oxygen consumption (approximately, 5 METS) for the elderly.
3. Monitor the patient’s ST-T changes and arrhythmias throughout exercise using an ultrasound monitor and measure blood pressure frequently.
4. Terminate exercise when the patient reaches the target heart rate or when any termination criterion is met; otherwise, continue up to the limit of tolerance of the patient.
5. Place the patient in the left lateral decubitus position immediately after exercise and record post-stress echocardiograms.
6. To obtain better quality images, it is desirable that whenever it is possible, the images should be recorded while the patient is holding his/her breath, although it may be quite difficult for the patient to do so because of post-exercise breathing difficulties.
7. It is desirable to complete echocardiographic recording within 2 min after the completion of exercise (Fig. 2).

**Fig. 2:**
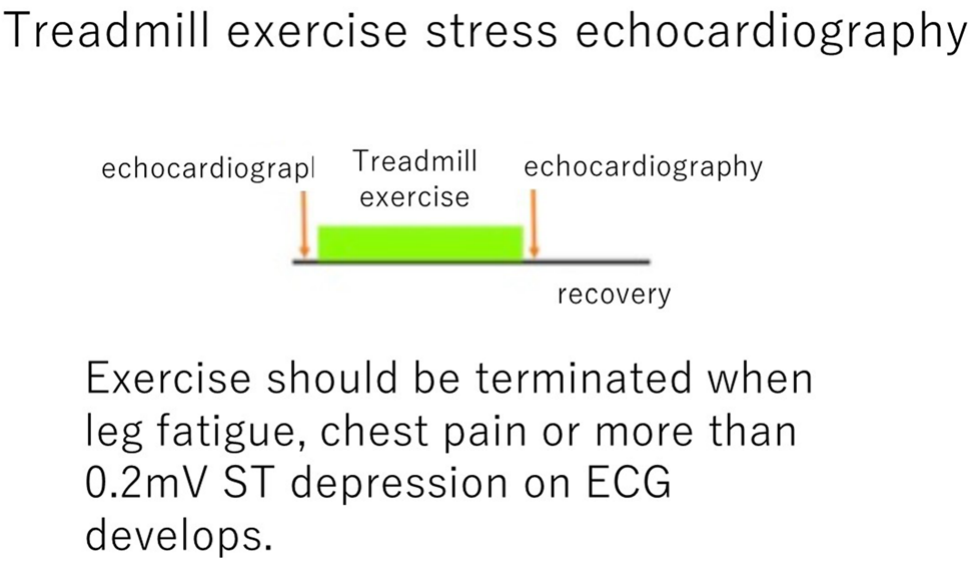
Exercise stress testing protocol (treadmill). The echocardiograms are obtained before and after the multistage stress test (Bruce protocol)
8. For several minutes after the completion of treadmill exercise, the patient will experience breathing difficulties, making clear imaging nearly impossible. The patient should be instructed in advance to exhale for as long as possible and inhale as briefly as possible immediately after the completion of exercise. The quality of ECGs can be improved by recording them during prolonged expiration by the patient. The ultrasound images are generally observable during exhalation by the patient, but not during inhalation. Concentrate on recording during exhalation because it is important to collect good-quality images; there is little value in attempting to acquire quality images while the patient is inhaling. If they cannot obtain adequate images despite the above effort, the attending staff may instruct the patient to hold his/her breath briefly. Limit recording of each view to 10 s or less and obtain images in multiple views within a short interval. Struggling with one view that cannot be visualized will lead to losing all recordings in the other views. It is recommended to complete all image recordings within 2 min after exercise. You need to quickly complete the procedure to acquire high-quality images that are adequate for the assessment, and this cannot be achieved without the patient’s co-operation.

#### Supine ergometer exercise stress

1. If the echo bed can be tilted laterally, place the patient in a slightly left lateral decubitus position and raise the upper portion so that the patient will assume a half-sitting position.
2. Record resting echocardiography, blood pressure, and ECGs.
3. Instruct the patient to place his/her feet on the pedals and commence the exercise. The pedaling rate should be set at 50–60 revolutions/min.
4. The workload is generally increased by 25 W every 3 min (Fig. 3a).

**Fig. 3:**
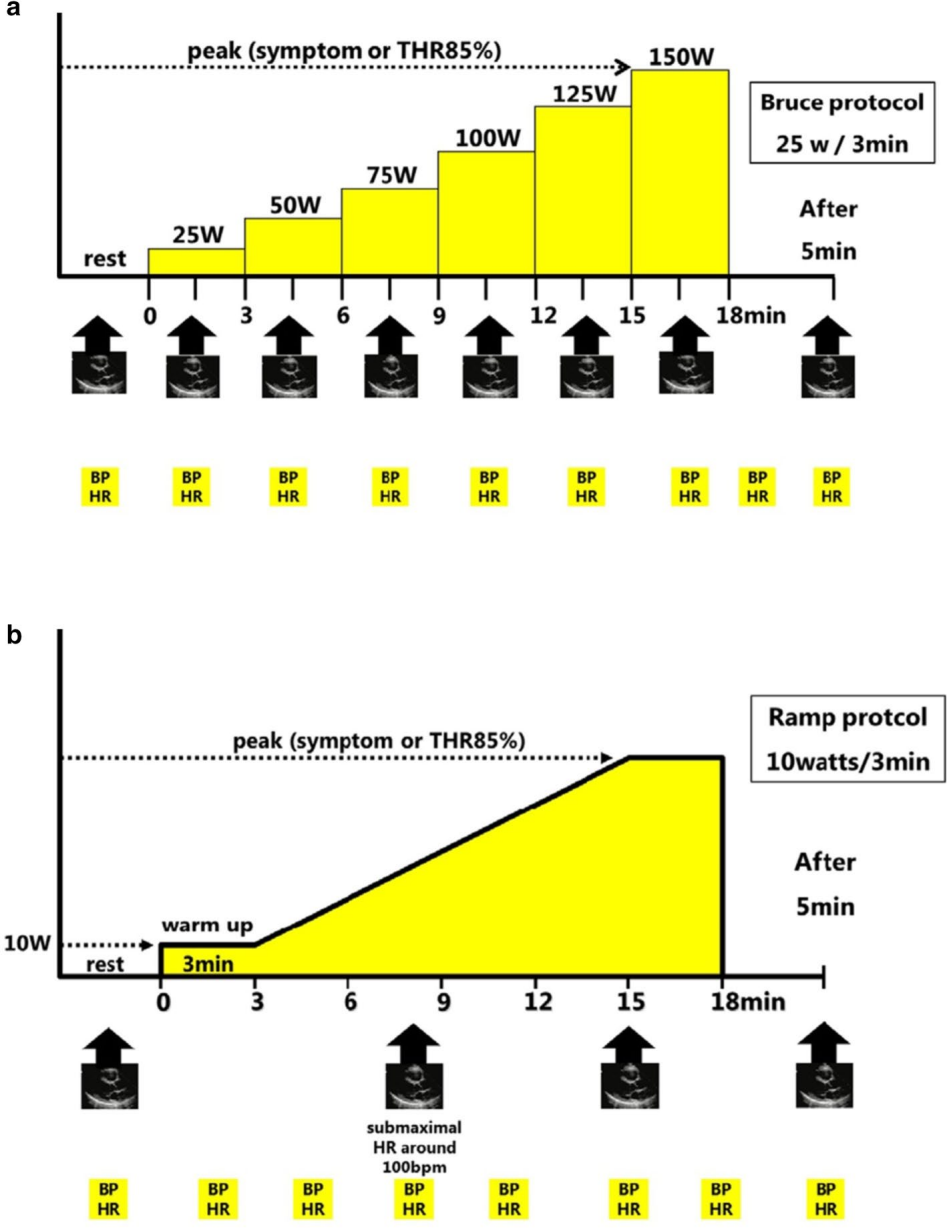
Exercise stress protocol (ergometer). **a** Multistage protocol: the workload is increased at 25-W increments every 3 min. **b** Ramp protocol: For the elderly with remarkable leg muscle weakness, a protocol with a 3-min warm-up at 10 W, followed by exercise with a 10-W increment every 3 min may be used. *BP* Blood pressure, *HR* heart rate, *THR* target heart rate
5. For the elderly with remarkable leg muscle weakness, a protocol using a 3-min warm-up at 10 W followed by 10 W increments every 3 min may be used (Fig. 3b).
6. Monitor the patient’s ST-T changes and arrhythmias throughout exercise using an ultrasound monitor and record an ECG and blood pressure every minute.
7. Terminate the exercise when the patient reaches the target heart rate, or when any termination criterion is met; otherwise, continue up to the limit of tolerance of the patient.
8. In the supine ergometer exercise stress testing echocardiography, echocardiographic assessment during exercise can be performed.
9. In the supine ergometer exercise stress testing, the images should be obtained with the patient placed in the left lateral decubitus position. Some of the authors experienced the following failures to acquire good-quality images at baseline; the improved data can be obtained once the test is commenced, i.e., after the patient starts pedaling with his/her feet on the pedals (with the feet raised). It is necessary to obtain data in the same views as those acquired at baseline. During the treadmill exercise testing, instruct the patient to exhale longer and inhale shorter than usual, and the operator should record as many images as possible during exhalation. In treadmill exercise echocardiography, image recording is commenced on completion of exercise after the patient is placed on the bed in a supine position. Stress-induced changes may resolve before being visualized unless imaging is done promptly; that is, image recording is under time constraints. On the other hand, in the supine ergometer exercise testing, the images can be recorded during exercise, which enables data collection over a longer period.

#### Handgrip exercise stress

Handgrip exercise is an isometric exercise that imposes a pressure load primarily on the left ventricle. Increasing exercise intensity markedly raises systolic pressure, while it only modestly affects heart rate. Numerous studies used a protocol with a 5-min sustained handgrip at 50% of the maximum voluntary contraction [31, 32]. Kerber et al. performed a handgrip exercise test and a treadmill exercise test in 90 patients with coronary artery disease to evaluate the ability of these tests to detect ischemia. During the treadmill testing, ST segment depression was observed in 25 patients, in contrast with just 3 patients with sustained handgrip exercise. The heart rate–systolic pressure product was 223 (×10^2^) for the former and small at 150 (×10^2^) for the latter; diastolic pressure was 93 mmHg for the latter versus 81 mmHg for the former [33]. The difference between the two modalities of exercise may be explained by the insufficient increase myocardial oxygen demand (less increase in heart rate) and the more coronary perfusion (due to increased diastolic blood pressure) in handgrip exercise. It was reported that the sensitivity of adenosine stress increased with handgrip exercise [34] and that strain evaluation of pre- and post-handgrip exercise images was useful in the diagnosis of myocardial ischemia [35].

#### Hyperventilation

Hyperventilation (30 bpm) is sustained for 6 min to induce coronary artery spasm [36].

#### Leg raising

Cardiac preload can be increased with leg raising during echocardiographic assessment. The patient is positioned supine with the leg raised at approximately 45° with the support of a third person or an object (e.g., chair) placed under the feet [37, 38]. In healthy individuals, leg raising increases the SV with a slight change in blood pressure. As an alternative to leg raising, the leg positive pressure method is available, in which the patient’s legs are pressurized by inflating cuffs placed around the legs using an automated air pump [39, 40].

### Stress test cessation criteria

Patient condition and blood pressure must be monitored while using ECG and during exercise. The test must be terminated immediately in the event of a remarkable increase or decrease in blood pressure or significant arrhythmias and appropriate measures should be taken. The criteria for terminating the testing based on the safety of patient are as follows:

1. Achievement of the target heart rate ([220 − patient’s age] × 0.85).
2. Excessive increase in blood pressure (systolic ≥ 220 mmHg, diastolic ≥ 120 mmHg).
3. Decrease in blood pressure (fall of ≥ 10 mmHg during exercise or no increase with continued exercise).
4. Manifestation of sustained tachyarrhythmia.
5. Manifestation of akinesis-like decrease in the wall motion or of wall motion decrease over a broad range involving the territory of more than one coronary artery.
6. ST segment depression of ≥ 0.2 mV on ECG.
7. Development or exacerbation of chest pain.
8. Leg exhaustion.
9. Development of any other symptoms that make continuation of exercise impossible.

### What to assess

The assessments are made at rest, during exercise, and post-exercise. Some parameters are unassessable during exercise. Attempts should be made to assess such parameters as promptly as possible after exercise. For items assessed during exercise, there is no consensus regarding when to make an assessment. We usually start echocardiographic observation immediately after the initiation of exercise and continue monitoring until a change can be observed, as the workload is gradually increased.

1. ECG and blood pressure.
2. Echocardiographic assessment.
  - In the parasternal long- and short-axis views (at the levels of the mitral valve, papillary muscles, and apex of the heart).
  - In the apical 4-chamber, 2-chamber, and long-axis views.
  - Of the LV inflow velocity patterns, mitral annular velocity patterns.
  - IOf the LVOT velocity patterns.
  - Of the tricuspid regurgitation (TR) velocity.
  - Severity assessments for each valvular disease.

### Precautions for implementation of exercise stress echocardiography

1. For the treadmill exercise testing, arrange the treadmill, a bed, and a compact echocardiographic system so that post-exercise imaging can be initiated as quickly as possible (Fig. 4).

**Fig. 4:**
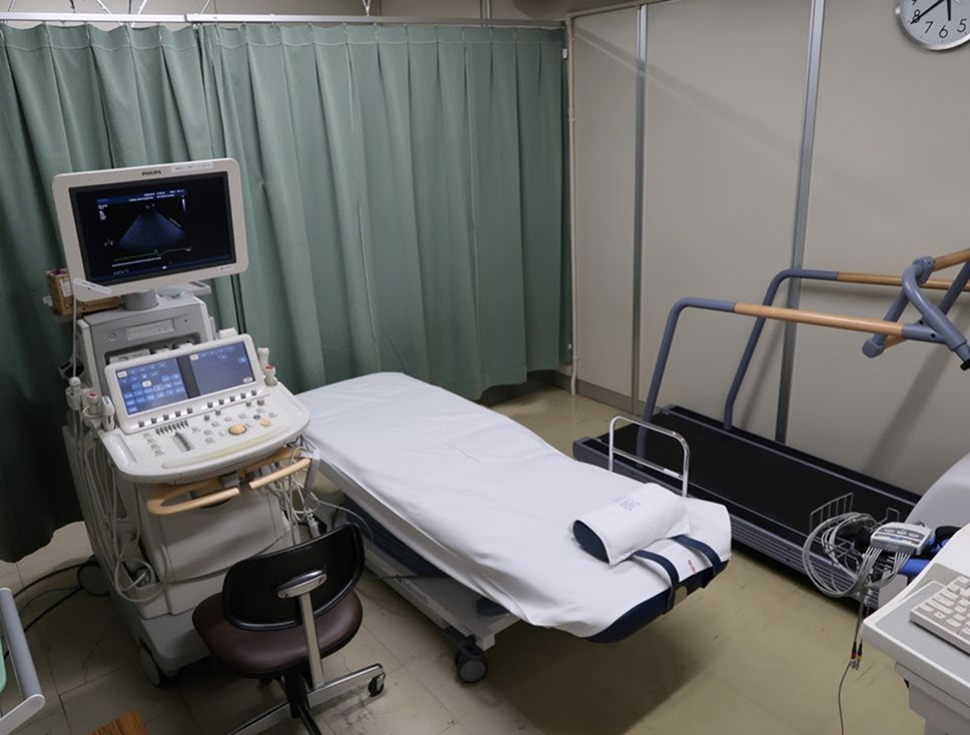
Treadmill exercise testing room (Kindai University Hospital). The treadmill, bed, and echocardiographic system should be arranged as compact as possible
2. Parasternal short-axis view, which contains the perfusion territories of all three coronary arteries, is critical in the exercise stress echocardiographic assessment of the wall motion; yet, oblique sections can be a cause of false-positive or false-negative results. An example: in the case of a long-axis view obtained by directing the beam through a lower intercostal space (Fig. 5a), the short-axis view gained by rotating this long-axis view by 90° results in an oblique section (Fig. 5b). In this section, the anteroseptal region is visualized close to the apex and the posterior wall more basally. Hence on visualizing the posterior wall, because of the influence of the mitral annulus, the inferior wall area may be misinterpreted as a site where there is decreased regional wall motion (arrow, Fig. 5c).

**Fig. 5:**
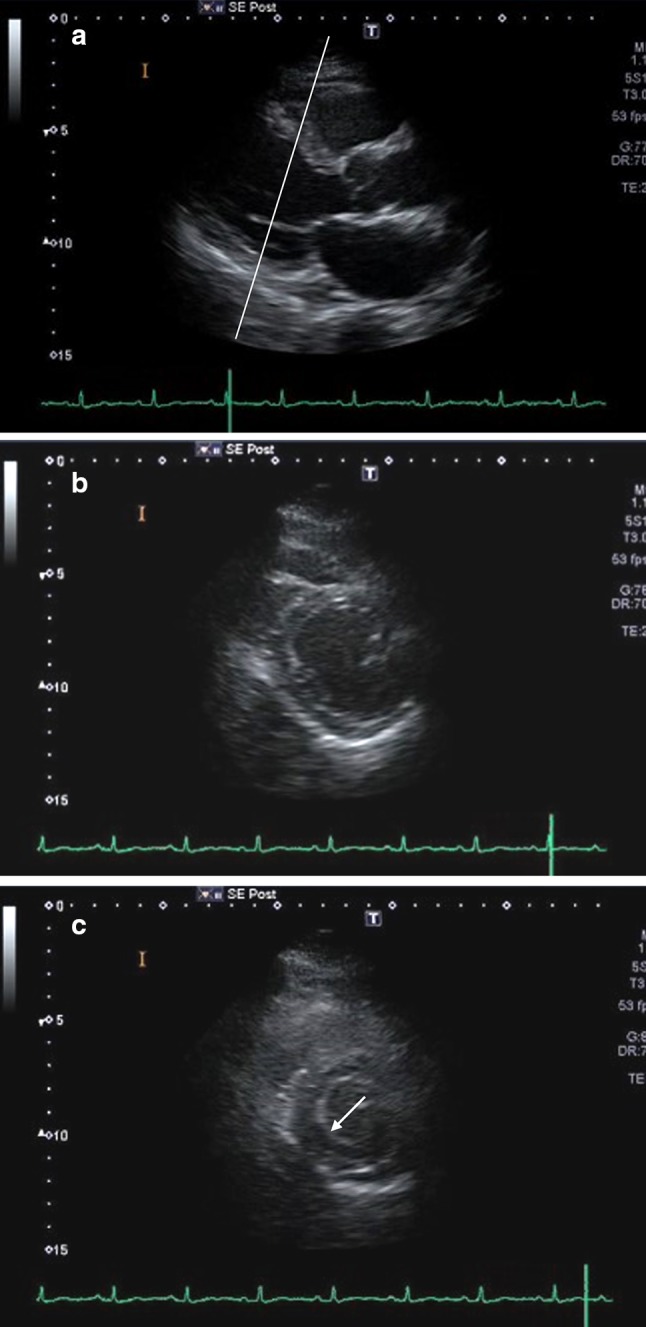
An example of an oblique view. **a** A long-axis view obtained by directing the beam through a lower intercostal space; **b** a short-axis view gained by rotating this long-axis view by 90°; **c** the resulting short-axis view is an oblique view with the anteroseptal region visualized close to the apex and the posterior wall more basally (arrow)
3. Exercise stress echocardiography is contraindicated in patients with unstable angina pectoris or symptomatic severe AS. The outpatient diagnosed as asymptomatic at the time of appointment for the test may possibly become symptomatic by the time of the test. It is important to reconfirm the absence of symptoms immediately before starting the test.
4. The examiner should be careful not to concentrate excessively on visualizing the structures, but to keep monitoring the symptoms, blood pressure, heart rate, and ECG of the patient during exercise to minimize the risk of cardiac events.
5. In the supine ergometer testing, the patient may suddenly become unable to pedal because of leg exhaustion at the peak load. Instruct the patient to tell the examiner at the timing when the exercise is becoming too hard to continue before starting the peak load stage; the examiner should start imaging slightly before the peak load stage. It is recommended, whenever possible, to monitor the rate of perceived exertion using the Borg Scale or another appropriate rating scale. If the patient has become completely unable to pedal, assistance may be provided. (It is more practical to commence imaging immediately after ergometer exercise is started and to continue the exercise test while changes in parameters are monitored.)
6. Care should be taken to perform imaging in coordination with the patient’s breathing. Although it may be difficult for the patient to hold a breath at peak load, attempts should be made to acquire the images while the patient is holding a breath whenever possible (forcing the patient to do so must be avoided). Regardless of such effort, images taken at peak load may not be of sufficient quality. Echocardiograms taken at a submaximal level (i.e., heart rate: approximately 100 bpm) may be used as a substitute for images at peak load.
7. The Images that can be obtained at peak load are limited. The examiner and the physician need to determine which images should be acquired at the peak load (e.g., transmitral and transtricuspid regurgitant flow velocity patterns, LVOT velocity patterns, images for wall motion assessment).

## Exercise stress echocardiographic measurements for heart diseases

### Ischemic heart disease

1. Wall motion.
  The assessment should be performed by reviewing the images at rest and exercise aligned side by side.
  The use of a 16-segment model of the left ventricle recommended by the American Society of Echocardiography [41]. Evaluate semiquantitatively and score the wall motion of each segment (see the Ischemic Heart Disease Report [appendix]). Grade the wall motion as normokinesis, hypokinesis, akinesis, or dyskinesis. Using too many grades should be avoided since it will lead to greater interobserver variability and decreased assessment objectivity. The wall motion assessment should be based on not only left endocardial excursion, but also changes in the wall thickening. The data from the systolic wall motion alone should be examined so as not to be misled by the diastolic wall motion. Normally, exercise stress increases the systolic wall motion and left ventricular (LV) wall thickness. With stress, the absence of an increase in wall motion or wall thickening is assessed as abnormal. Exacerbation of wall motion of a normal or hypokinetic wall segment by one grade indicates a positive result for ischemia. The sufficient echocardiogram qualities facilitate diagnosis and contribute to the diagnostic accuracy. Constant effort is required to achieve the best possible imaging quality, using optimal gain and slice plane selection.

### Valvular heart disease

#### Mitral stenosis

Exercise stress echocardiography is employed in cases where resting echocardiographic data are not commensurate with the severity of symptoms, i.e., asymptomatic MS despite severe stenosis, or symptomatic MS despite mild to moderate disease.

1. Transmitral pressure gradient.
  A mean pressure gradient is assessed based on mitral flow velocity at rest and exercise. An estimated mean pressure gradient of ≥ 15 mmHg during exercise indicates severe MS [42].
2. Exercise-induced PH.
  Transtricuspid pressure gradient is assessed at rest and during exercise. An estimated systolic pulmonary artery pressure (PAP) of ≥ 60 mmHg during exercise indicates severe MS [42].

#### Mitral regurgitation

1. Severity of MR.
  Changes in MR are assessed quantitatively or semiquantitatively, comparing rest and exercise. It has been shown that worsening MR with exercise stress is associated with a poor prognosis [43]. The assessment of MR severity has been reported to be impossible in patients with heart rate ≥ 115 bpm [44].
2. Exercise-induced PH.
  Transtricuspid pressure gradient at rest and exercise is assessed. An estimated systolic pulmonary artery pressure of ≥ 60 mmHg during exercise suggests exercise-induced PH [45].
3. Contractile reserve.
  A decline in LV reserve (a change in LVEF during exercise ≤ 5% [46] or a change in the global longitudinal strain ≤ 2% [47]) and reduction in right ventricular systolic function (tricuspid annular plane systolic excursion < 18 mm [48]) have been reported to be predictors of a poor prognosis and of decreased cardiac function following mitral valve surgery.

#### Aortic stenosis

1. Aortic flow velocity and mean pressure gradient.
  Changes in the aortic flow velocity, mean pressure gradient, and CO (or pulsed Doppler time–velocity integral across the LVOT as a surrogate) are assessed, comparing at rest and exercise. A ≥ 18 mmHg increase in the mean pressure gradient at exercise has been reported to be associated with a poor prognosis, showing the usefulness of this evaluation in risk stratification of asymptomatic patients with AS [49].
2. Aortic valve area.
  The aortic valve area (AVA) is assessed under conditions of increased CO with stress.
3. Exercise-induced PH.
  Transtricuspid pressure gradient is assessed at rest and during exercise. An estimated systolic PAP of ≥ 60 mmHg during exercise suggests exercise-induced PH [50].

#### Aortic regurgitation (AR)

1. Contractile reserve.
  In patients with asymptomatic severe AR, reduced contractile reserve (increase in LVEF during exercise ≤ 5%) has been reported to be a predictor of future cardiac functional deterioration and of postoperative cardiac function [51].

### Cardiomyopathy

#### Dilated cardiomyopathy

1. Contractile reserve.
  Contractile reserve has been reported to well predict prognosis and beta blockers response in dilated cardiomyopathy. The stress protocols and assessment methods vary from study to study. Contractile reserve can be assessed based on low-dose (10–20 μg/kg/min) dobutamine-induced changes in LVEF [52], LV volume [53], and strain [54] from the resting state.
2. Functional MR.
  Functional MR at rest and exercise is assessed visually, or if possible, quantitatively. An increase in functional MR with stress (effective regurgitant orifice ≥ 13 mm^2^) has been reported to predict a poor prognosis [55].
3. Exercise-induced PH.
  Transtricuspid pressure gradient is assessed at rest and exercise. An increase in estimated systolic PAP of ≥ 60 mmHg with exercise has been reported to indicate a poor prognosis [56].
4. Prediction of cardiac resynchronization therapy (CRT).
  LVEF response to low-dose dobutamine infusion (10 μg/kg/min, LVEF ≥ 7.5%) has been reported to be useful in the prediction of responders to CRT [57].

#### Hypertrophic cardiomyopathy

1. LVOT obstruction.
  LVOT obstruction may appear at exercise, but not at rest in some patients (exercise-induced LVOT obstruction; maximum LVOT gradient ≥ 50 mmHg). Exercise stress echocardiography is indicated in the diagnosis of such patients [58].
2. Wall motion assessment.
  Patients without significant coronary stenosis may have relative subendocardial ischemia in the hypertrophic myocardium, and in such patients wall motion abnormalities may be induced by exercise stress. LV wall motion abnormalities with exercise have been reported to indicate a poor prognosis in hypertrophic cardiomyopathy [59].
3. MR.
  Changes in MR that are associated with systolic anterior motion of the mitral valve are also assessed [60].
4. Exercise-induced PH.
  Exercise-induced PH, which is often a complication of LV diastolic dysfunction, is assessed [61].

### Ph

1. Exercise-induced PH.
  Transtricuspid pressure gradient at rest and exercise is assessed. An estimated systolic PAP of ≥ 40–50 mmHg, depending on the exercise stress testing method used, indicating exercise-induced PH [62].
2. CO.
  Since PAP depends on CO, CO is evaluated at rest and exercise. An increase by 3.0 mmHg/L/min in mean PAP (mPAP) as a function of CO (∆mPAP/∆CO) in healthy subjects has been reported [63].^5^

## Dobutamine stress echocardiography (for reference)

### Introduction

Currently, dobutamine echocardiography is not covered by the national health insurance in Japan, whereas in actual clinical settings there are numerous patients, including the elderly, who are unable to perform exercise stress testing and for whom pharmacological stress echocardiography is deemed necessary. In addition, for diagnosis of ischemic heart disease (IHD), pharmacological stress echocardiography is less costly than radionuclide scanning and can be conducted, unlike contrast-enhanced computed tomography or magnetic resonance imaging, in patients with renal dysfunction, bronchial asthma, or hypersensitivity to contrast media. Although this guideline focuses on exercise stress echocardiography, dobutamine stress echocardiography is also described herein for reference, considering the clinical utility of this modality.

### Objectives of dobutamine stress echocardiography

A considerable number of elderly patients and patients with orthopedic or neurological disease are unable to perform exercise stress testing of sufficient intensity. In such cases, dobutamine echocardiography is indicated. This modality, which permits stable tomographic image recording during stress, allows for more detailed assessment of LV wall motion, compared with exercise stress echocardiography. Dobutamine stress echocardiography is useful not only in the evaluation of myocardial ischemia, but also in the assessment of myocardial viability and contractile reserve in people following myocardial infarction or those with chronic IHD. Furthermore, in distinguishing true, severe AS from ‘pseudo-severe’ AS in those with low-flow, low-gradient severe AS with reduced LVEF.

### Safety of dobutamine stress echocardiography

The most commonly reported complications of dobutamine stress echocardiography include decreased blood pressure (1.7%), supraventricular tachycardia (1.3%), increased blood pressure (1.3%), atrial fibrillation (0.9%), atrioventricular block (0.23%), ventricular tachycardia (0.15%), coronary artery spasm (0.14%), ventricular fibrillation (0.04%), myocardial infarction (0.02%), cerebrovascular disorder (0.005%), and cardiac rupture (0.002%) [65, 66]. In performing dobutamine stress echocardiography, we recommend equipping the testing room with an emergency cart with supplies ready for use, to enable the attending staff to take necessary measures promptly when any of these complications occur. Palpitations, sensation of warmth, and voiding sensation are relatively frequently observed, but rapidly subside when dobutamine infusion is terminated. If any of these symptoms develop during testing, the attending staff should explain this to the patient and continue the test providing the event is deemed to be tolerable. Other possible complications include bradycardia and a decline in blood pressure due to vagal reflexes; in the event of these complications, if discussion or handgrip exercise does not improve the situation, atropine should be administered. In addition, it is recommended to keep the patient at rest and observe his/her clinical course for at least 30 min post-stress.

### Implementation of dobutamine stress echocardiography

Dobutamine infusion in patients with IHD should be initiated at 5 μg/kg/min, with an increment to 10, 20, 30, and 40 μg/kg/min at 5-min intervals. In cases where dobutamine doses up to 40 μg/kg/min neither achieve the target heart rate nor meet any cessation criterion, the dose should be increased to 50 μg/kg/min, or 0.25 mg of atropine should be administered every minute up to 2 mg at maximum. It is also helpful to add a handgrip exercise stress. Low-dose dobutamine stress testing for assessment of myocardial viability or AS should be initiated at 5 μg/kg/min, with a 5-μg increment every 5 min to 10, 15, and 20 μg/kg/min.

Criteria for dobutamine stress test termination include the following:

1. The target heart rate of (220 − age) × 0.85 (bpm) has been reached.
2. A significant decrease in blood pressure (systolic: ≤ 80 mmHg) or significant increase (systolic: ≥ 220 mmHg).
3. Manifestation of a significant tachyarrhythmia.
4. Manifestation of intolerable chest pain, nausea, headache, urge to void, etc.
5. Manifestation of severe myocardial ischemia, such as a new akinesis-like decrease in wall motion and deteriorating wall motion over a broad area involving the territory of more than one coronary artery.
6. ST segment depression of ≥ 0.2 mV on ECG.

### Dobutamine echocardiographic measurements for heart diseases

#### Ischemic heart disease

*Wall motion* As in the case of exercise stress echocardiography, wall motion is graded as normokinesis, hypokinesis, akinesis, or dyskinesis. Responses to dobutamine stress are categorized into the following four types: (1) improvement: wall motion improves with low-dose dobutamine as well as with high doses; (2) biphasic: wall motion improves with low-dose dobutamine but worsens with high doses; (3) worsening: wall motion decreases with low-dose stress and worsens with high doses; and (4) fixed: wall motion remains unchanged with dobutamine stress. Improvement indicates myocardial viability without ischemia. The biphasic or worsening responses are seen in the presence of both myocardial viability and ischemia. The fixed type, where wall motion remains akinetic during low- and high-dose dobutamine stress, indicates a lack of myocardial viability.

#### AS

The peak velocity and pressure gradient of aortic flow depend on the flow rate, i.e., the volume of blood that flows past the aortic orifice per unit time. The flow rate is obtained from stroke volume (SV) divided by ejection time. Generally, an indexed SV (SV divided by the body surface area) < 35 mL/m^2^ is defined as ‘low flow,’ and a mean aortic pressure gradient < 40 mmHg as ‘low gradient’. Low-flow, low-gradient severe AS despite the aortic valve area (AVA) satisfying the diagnostic criteria for severe AS suggests either decreased LVEF or decreased stroke volume due to small LV end-diastolic volume with normal LVEF in the absence of significant MR. The former is referred to as classical low-flow low-gradient severe AS, and the latter as paradoxical low-flow low-gradient severe AS.

Classical low-flow low-gradient severe AS can be divided into two clinical states: true, severe AS and ‘pseudo-severe’ AS, where complete opening of the aortic valve is hindered, despite AS of intermediate or mild intensity, by decreased LVEF. Dobutamine echography is useful in differentiating between these two states.

True, severe AS is defined by an AVA ≤ 1.0 cm^2^ with a peak aortic valve velocity ≥ 4.0 m/s and a mean aortic valve gradient  40 mmHg [67].

Pibarot et al. [68]. define ‘true severe AS’ using the following criteria, in addition to ≥ 20% increase in SV with 20 μg/kg/min dobutamine infusion; all other AS cases are considered ‘pseudo-severe.’

1. An increase in mean pressure gradient to > 30 mmHg.
2. Peak-stress effective orifice area ≤ 1.0 cm^2^ or < 1.2 cm^2^, depending on testing conditions.
3. An increase in effective orifice area of < 0.3 cm^2^.

In other words, where complete valve opening appears to occur with increased CO, this is diagnosed as ‘pseudo-severe’ AS.

**Fig. 6:**
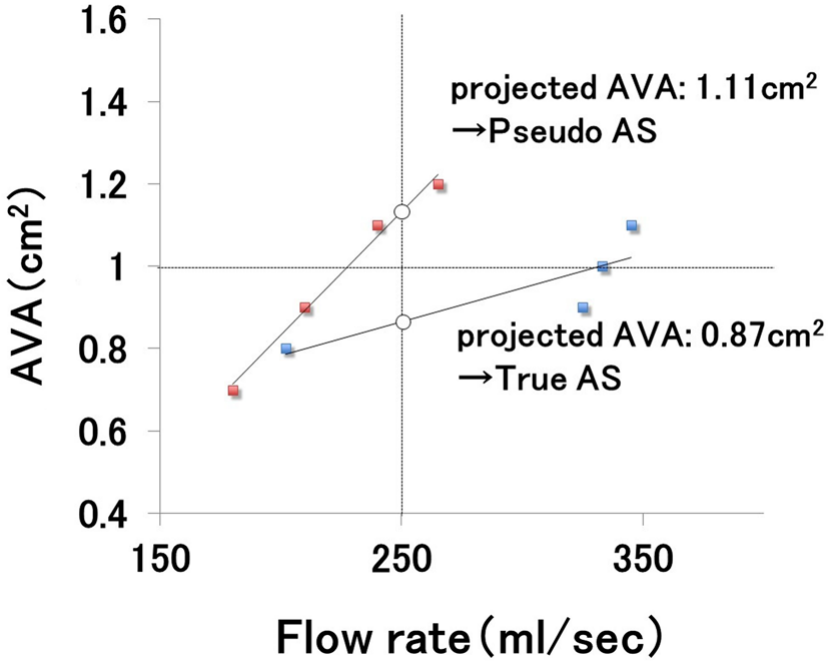
Calculation of the projected AVA. AVA (cm^2^) is plotted against flow rate (mL/s) at four stages (at rest and with dobutamine stress at 5, 10, and 20 μg/kg/min) to generate a linear regression formula. The AVA at flow rate of 250 mL/s is obtained from this formula as the projected AVA. If this value is < 1.0 cm^2^, true severe AS is diagnosed

Cases showing an insufficient (< 20%) SV increase with dobutamine stress are considered to lack LV contractile reserve, and in these circumstances, differentiation between true and pseudo-severe AS is impossible. Patients lacking LV contractile reserve have been reported to be at higher risk of operational death compared to those with reserve, but to be associated with a better prognosis following aortic valve replacement surgery than with medical treatment [68]. In addition to the above semiquantitative assessment method, a method based on the projected AVA [69] is available. AVA (cm^2^) is plotted against flow rate (mL/s) at four stages (at rest and with dobutamine stress at 5, 10, and 20 μg/kg/min) to generate a linear regression formula, and using this formula the AVA at a flow rate of 250 mL/s is calculated as the projected AVA. AS is diagnosed as truly severe if the projected AVA is less than 1.0 cm^2^ (Fig. 6).

## Explanation and consent form for stress echocardiography

### Why we need to test your heart

Stress echocardiography by putting stress on your heart is required to uncover hidden conditions that are not detectable with resting echocardiography, to allow more detailed assessment of how sick you are.

### Objectives of testing

The objectives differ depending on the type of heart disease you have.

1. *Coronary artery disease* To see whether left ventricular wall motion abnormalities appear with stress and to assess whether blood supply from the arteries of the heart to the heart muscle is sufficient.
2. *Valvular heart disease* To diagnose how heart function changes during exercise and to decide whether surgery is necessary or not.
3. *Cardiomyopathy* To detect heart reserve and changes in blood flow during exercise, and to select medications and treatment of the condition

### Testing methods

We put stress on the heart using either exercise or drug stimulation. For exercise stress testing, the various methods are shown below. Electrocardiograms (ECGs) will be obtained before stress and during or after stress. A cardiologist will be present during the tests to ensure your safety and monitor your clinical condition. Your ECGs and blood pressure will be recorded. In the testing room, emergency equipment and drugs are readily available for the staff to provide urgent measures when any undesirable event occurs.

- Exercise stress echocardiography.
  - Treadmill.
  - Cycle ergometer (sitting, lying).
  - Handgrip.
  - Six-minute walk.
  - Master step test.
- Pharmacological (dobutamine) stress echocardiography.

### Explanation of each method

#### Treadmill exercise stress test

You will be asked to walk on a moving belt, which looks like a conveyor belt, at a speed set by the machine. The grade and speed will gradually be increased every 2–3 min. A cardiologist will be supervising the test for your safety and from time to time may ask you how you are feeling. Your ECG and blood pressure will be monitored and recorded during the test. The test will be stopped if your heart rate has reached the target level, or if you no longer feel able to exercise because of breathlessness, chest pain, or any other reason, or if any abnormality is found on your ECG. Echocardiography will be performed before and after exercise.

#### Ergometer stress test

You will be asked to pedal in a sitting or lying position at a constant rate. The pedaling rate suitable for you will be determined for the test. The resistance you will feel will be mild in the beginning and will increase gradually, but you are asked to continue pedaling at the same rate. A cardiologist will supervise the test for your safety and from time to time may ask you how you are feeling. Your ECG and blood pressure will be monitored and recorded during the test. The test will be stopped if your heart rate has reached the target level, or if you no longer feel able to exercise because of breathlessness, chest pain, or any other reason, or if any abnormality is found on your ECG. Echocardiography will be performed before and and during after exercise.

#### Handgrip exercise stress test

You will be asked to continue grasping a handgrip for 4–5 min at about half of your maximum grip strength. If you notice any symptoms during the exercise, please let the attending staffs know. If you no longer feel able to continue the exercise, the test will be stopped at that point. Echocardiography will be performed before, and during or after exercise.

#### Six-minute walk test

You will be asked to walk for 6 min at your own pace on a predetermined course with an attending examiner, who will check on you if you are short of breath or have chest pain along the way. Echocardiography will be performed before and after exercise.

#### Master step test

This is an exercise test where you step up and down, and continue this for either ninety seconds or three minutes, as predetermined by a cardiologist. The number of steps to be used depends on your age and gender. You will be instructed to step up and down the Master step. The test will be stopped if you no longer feel able to continue the exercise because of breathlessness or chest pain, or if any abnormality is noted on your ECG. Echocardiography will be performed before and after exercise.

#### Pharmacological (dobutamine) stress echocardiography

This is a test to diagnose your heart reserve by imposing stress on your heart with drug infusion. Dobutamine (a drug that stimulates the heart muscle) is used. Symptoms such as a rapid heartbeat and flushing of the face are expected to occur but will disappear within 10 min after the end of the infusion.

### Risks, complications, and side effects

There are small risks associated with exercise stress echocardiography. Specifically, these risks include lowering or raising of blood pressure, dizziness, an irregular pulse, fainting, and chest discomfort. Stress imposed on the heart for diagnostic purposes may cause lengthy angina pain, or even myocardial infarction or arrhythmias but serious complications are rare. In the event of a side effect, emergency measures including urgent hospitalization may be required (urgent hospitalization is needed in one in about 43,000 tests; death can occur once in about 264,000 tests [source: Japanese Society of Electrocardiography])—medical costs will then need to be borne by the patient, as is usually the case with our healthcare services. During treadmill stress testing, a fall and resulting bone fracture may occur. If the patient’s legs have become very tired during the exercise, the examiner may stop the machine right away to prevent accidents such as falling off the treadmill.

Pharmacological stress testing is also reported to be associated with complications, as is the case with exercise stress testing. Stress testing with drug stimulation is not necessarily safer.

Being a test that imposes stress on the heart, stress echocardiography might result in the development of heart attack or arrhythmias, or very rarely, death. Rates of occurrence of emergency hospitalization or serious but non-fatal complications such as myocardial infarction are estimated at 0.2% or less.

I have received an explanation of the testing described above and satisfactory answers to the questions I have asked; I agree to engage in a stress test.

(I understand that this consent can be withdrawn at any time.)

Left cardiac system standard measurements

**Table:**

	Rest	Sub maximal	Peak	Post Ex
Heart rate	/min	/min	/min	/min
LAD	mm	mm	mm	mm
LVDd	mm	mm	mm	mm
LVDd index	mm/m^2^	mm/m^2^	mm/m^2^	mm/m^2^
LVDs	mm	mm	mm	mm
LVDs index	mm/m^2^	mm/m^2^	mm/m^2^	mm/m^2^
%FS	%	%	%	%
LA volume index	ml/m^2^	ml/m^2^	ml/m^2^	ml/m^2^
LVEDV biplane	ml	ml	ml	ml
LVESV biplane	ml	ml	ml	ml
SV bipalne	ml	ml	ml	ml
EF biplane	%	%	%	%

Doppler measurements

**Table:**

	Rest	Sub maximal	Peak	Post Ex
Heart rate	/min	/min	/min	/min
E wavw velocity	cm/s	cm/s	cm/s	cm/s
DcT	ms	ms	ms	ms
E/A				
E′ sep	cm/s	cm/s	cm/s	cm/s
S′ sep	cm/s	cm/s	cm/s	cm/s
E′ lat	cm/s	cm/s	cm/s	cm/s
S′ lat	cm/s	cm/s	cm/s	cm/s
E′ average	cm/s	cm/s	cm/s	cm/s
E/E′ averege				

Right cardiac system standard measurements

**Table:**

	Rest	Sub maximal	Peak	Post Ex
Heart rate	/min	/min	/min	/min
RV EDA	cm^2^	cm^2^	cm^2^	cm^2^
RV ESA	cm^2^	cm^2^	cm^2^	cm^2^
FAC	%	%	%	%
E′ RV	cm/s	cm/s	cm/s	cm/s
S′ RV	cm/s	cm/s	cm/s	cm/s
TAPSE	mm	mm	mm	mm
TRPG	mmHg	mmHg	mmHg	mmHg

Valvular heart disease report

**Table:**

MS	Rest	Sub maximal	Peak	Post Ex
HR	bpm	bpm	bpm	bpm
MV area CW	cm^2^	cm^2^	cm^2^	cm^2^
Peak velocity	cm/s	cm/s	cm/s	cm/s
Max PG	mmHG	mmHG	mmHG	mmHG
Mean PG	mmHG	mmHG	mmHG	mmHG
PHT	ms	ms	ms	ms

MR	Rest	Sub maximal	Peak	Post Ex
MR RV (volumetric)	ml	ml	ml	ml
MR RF	%	%	%	%
MR ERO (volumetric)	cm^2^	cm^2^	cm^2^	cm^2^
MR RV (PISA)	ml	ml	ml	ml
MR ERO (PISA)	cm^2^	cm^2^	cm^2^	cm^2^
Vena contracta	mm	mm	mm	mm
PHT	ms	ms	ms	ms

AS	Rest	Sub maximal	Peak	Post Ex
AVA	cm^2^	cm^2^	cm^2^	cm^2^
AVAi	cm^2/^m^2^	cm^2/^m^2^	cm^2/^m^2^	cm^2/^m^2^
VTI AV	cm	cm	cm	cm
VTI ration (AV/LVOT)				
HR	/min	/min	/min	/min
Ejection time	ms	ms	ms	ms
Peak velocity	cm/s	cm/s	cm/s	cm/s
Mean PG	mmHG	mmHG	mmHG	mmHG

AR	Rest	Sub maximal	Peak	Post Ex
AR RV (volumetric)	ml	ml	ml	ml
AR RF	%	%	%	%
AR ERO (volumetric)	cm^2^	cm^2^	cm^2^	cm^2^
AR RV (PISA)	ml	ml	ml	ml
AR ERO (PISA)	cm^2^	cm^2^	cm^2^	cm^2^
Vena contracta	mm	mm	mm	mm
PHT	ms	ms	ms	ms

Pulmonary hypertension report

**Table:**

	Rest	Sub maximal	Peak	Post Ex
BP	mmHG	mmHG	mmHG	mmHG
HR	/min	/min	/min	/min
TR peak velocity	cm/s	cm/s	cm/s	cm/s
TRPG	mmHG	mmHG	mmHG	mmHG
RVSP	mmHG	mmHG	mmHG	mmHG
LVOT diameter	mm			
LVOT TVI	cm	cm	cm	cm
HR	/min	/min	/min	/min
Cardiac output	l/min	l/min	l/min	l/min
Stroke volume	ml	ml	ml	ml
RVOT diameter	mm	mm	mm	mm
RVOT TVI	cm	cm	cm	cm
HR	/min	/min	/min	/min
RV cardiac output	l/min	l/min	l/min	l/min
RV stroke volume	l/min/m^2^	l/min/m^2^	l/min/m^2^	l/min/m^2^

## Footnotes

1 Statement on exercise echocardiography for ischemic heart disease from the American Heart Association (AHA) Guideline. The 2003 AHA guideline [6] includes a new Class I recommendation: Exercise echocardiography for diagnosis of myocardial ischemia in intermediate-risk patients on digoxin, with LV hypertrophy, or with more than 1 mm ST depression at rest on the baseline electrocardiographic assessment; and patients with Wolff–Parkinson–White syndrome or a conduction disturbance such as left bundle-branch block. Namely, exercise echocardiography combined with wall motion assessment should be performed in patients showing abnormal waveforms on the 12-lead electrocardiogram at rest.

2 As the tricuspid regurgitation pressure gradient increases during exercise with age, even in healthy individuals, caution is required for the interpretation of the results of evaluation, especially in the elderly [30].

3 Exercise stress testing is contraindicated in patients with symptomatic severe AS. The patient diagnosed as asymptomatic at the time of appointment for the test may present with symptoms at the time of testing. It is imperative to reconfirm the absence of symptoms immediately before testing. Pharmacological stress should be selected for patients incapable of performing exercise stress testing because of concomitant peripheral blood, orthopedic, or neurological disease. Patients incapable of understanding the explanation on giving consent to testing because of dementia or other reasons should be excluded from exercise stress echocardiography.

4 In treadmill exercise stress testing, patients may not be able to keep up with the machine’s walking speed. With elderly patients, due caution should be exercised to avoid the risk of falls, while ensuring that the treadmill belt velocities to be used are suitable for the patient and are reset if necessary before testing.

5 mPAP was estimated as mPAP = 0.6 × estimated systolic PAP + 2 (mmHg) [64].

## References

[1] Pellikka PA, Nagueh SF, Elhendy AA (2007). American Society of Echocardiography recommendations for performance, interpretation, and application of stress echocardiography. J Am Soc Echocardiogr.

[2] Lancellotti P, Pellikka PA, Budts W (2016). The clinical use of stress echocardiography in non-ischaemic heart disease: recommendations from the European Association of Cardiovascular Imaging and the American Society of Echocardiography. Eur Heart J Cardiovasc Imaging.

[3] Schinkel A, Bax JJ, Geleijnse ML (2003). Noninvasive evaluation of ischaemic heart disease: myocardial perfusion imaging or stress echocardiography?. Eur Heart J.

[4] Hansen JE (1984). Exercise instruments, schemes, and protocols for evaluating the dyspneic patient. Am Rev Respir Dis.

[5] Myers J, Arena R, Franklin B (2009). Recommendations for clinical exercise laboratories: a scientific statement from the American Heart Association. Circulation.

[6] Cheitlin MD (2003). ACC/AHA/ASE 2003 guideline update for the clinical application of echocardiography: summary article: a report of the American College of Cardiology/American Heart Association Task Force on Practice Guidelines (ACC/AHA/ASE Committee to Update the 1997 Guidelines for the Clinical Application of Echocardiography). Circulation.

[7] Banerjee A, Newman DR, Van den Bruel A (2012). Diagnostic accuracy of exercise stress testing for coronary artery disease: a systematic review and meta-analysis of prospective studies. Int J Clin Pract.

[8] de Jong MC, Genders TS, van Geuns RJ (2012). Diagnostic performance of stress myocardial perfusion imaging for coronary artery disease: a systematic review and meta-analysis. Eur Radiol.

[9] Heijenbrok-Kal MH, Fleischmann KE, Hunink MG (2007). Stress echocardiography, stress single-photon-emission computed tomography and electron beam computed tomography for the assessment of coronary artery disease: a meta-analysis of diagnostic performance. Am Heart J.

[10] Metz LD, Beattie M, Hom R (2007). The prognostic value of normal exercise myocardial perfusion imaging and exercise echocardiography: a meta-analysis. J Am Coll Cardiol.

[11] Roger VL, Pellikka PA, Bell MR (1997). Sex and test verification bias: impact on the diagnostic value of exercise echocardiography. Circulation.

[12] Arruda A, Das F, Roger R (2001). Prognostic value of exercise echocardiography in 2632 patients 65 years of age. J Am Coll Cardiol.

[13] Garrido IP, Peteiro J, Garcia-Lara J (2005). Prognostic value of exercise echocardiography in patients with diabetes mellitus and known or suspected coronary artery disease. Am J Cardiol.

[14] Elhendy A, Mahoney DW, Burger KN (2004). Prognostic value of exercise echocardiography in patients with classic angina pectoris. Am J Cardiol.

[15] Ryan T, Armstrong WF, O’Donnel JA (1987). Risk stratification following acute myocardial infarction during exercise two-dimensional echocardiography. Am Heart J.

[16] Quintana M, Lindvall K, Ryden L (1995). Prognostic value of predischarge exercise stress echocardiography after acute myocardial infarction. Am J Cardiol.

[17] Peteiro J, Monserrat L, Vazquez E (2003). Comparison of exercise echocardiography to exercise electrocardiographic testing added to echocardiography at rest for risk stratification after uncomplicated acute myocardial infarction. Am J Cardiol.

[18] Arruda A, McCully R, Oh J (2001). Prognostic value of exercise echocardiography in patients after coronary artery bypass surgery. Am J Cardiol.

[19] Shaw LJ, Eagle KA, Gersh BJ (1996). Meta-analysis of intravenous dipyridamole-thallium-201 imaging (1985–1994) and dobutamine echocardiography (1991–1994) for risk stratification before vascular imaging. J Am Coll Cardiol.

[20] Lalka SG, Sawada SG, Dalsing MC (1992). Dobutamine stress echocardiography as a predictor of cardiac events associated with aortic surgery. J Vasc Surg.

[21] Lane RT, Sawada SG, Segar DS (1991). Dobutamine stress echocardiography for assessment of cardiac risk before noncardiac surgery. Am J Cardiol.

[22] Eichelberger JP, Schwarz KQ, Black ER (1993). Predictive value of dobutamine echocardiography just before noncardiac vascular surgery. Am J Cardiol.

[23] Hecht H, DeBord L, Shaw R (1993). Usefulness of supine bicycle stress echocardiography for detection of restenosis after percutaneous transluminal coronary angioplasty. Am J Cardiol.

[24] Kafka H, Leach A, Fitzgibbon G (1995). Exercise echocardiography after coronary artery bypass surgery: correlation with coronary angiography. J Am Coll Cardiol.

[25] Hecht HS, DeBord L, Sotomayor N (1993). Supine bicycle stress echocardiography: peak exercise imaging is superior to postexercise imaging. J Am Soc Echocardiogr.

[26] Pierard LA, De Landsheere CM, Berthe C (1990). Identification of viable myocardium by echocardiography during dobutamine infusion in patients with myocardial infarction after thrombolytic therapy: comparison with positron emission tomography. J Am Coll Cardiol.

[27] Applegate RJ, Dell’Italia LJ, Crawford MH (1987). Usefulness of two dimensional echocardiography during low-level exercise testing early after uncomplicated myocardial infarction. Am J Cardiol.

[28] Lancellotti P, Hoffer EP, Pierard LA (2003). Detection and clinical usefulness of a biphasic response during exercise echocardiography early after myocardial infarction. J Am Coll Cardiol.

[29] Bergeron S, Ommen S, Bailey K (2004). Exercise echocardiographic findings and outcome of patients referred for evaluation of dyspnea. J Am Coll Cardiol.

[30] Kovacs G, Berghold A, Scheidl S (2009). Pulmonary arterial pressure during rest and exercise in healthy subjects: a systematic review. Eur Respir J.

[31] Voci P, Testa G, Plaustro G (1999). Coronary Doppler intensity changes during handgrip: a new method to detect coronary vasomotor tone in coronary artery disease. J Am Coll Cardiol.

[32] Kivowitz C, Parmley WW, Donoso R (1971). Effects of isometric exercise on cardiac performance. The grip test. Circulation.

[33] Kerber RE, Miller RA, Najjar SM (1975). Myocardial ischemic effects of isometric, dynamic and combined exercise in coronary artery disease. Chest.

[34] Tawa CB, Baker WB, Kleiman NS (1996). Comparison of adenosine echocardiography, with and without isometric handgrip, to exercise echocardiography in the detection of ischaemia in patients with coronary artery disease. J Am Soc Echocardiogr.

[35] Ryo K, Tanaka H, Kaneko A (2012). Efficacy of longitudinal speckle tracking strain in conjunction with isometric handgrip stress test for detection of ischemic myocardial segments. Echocardiography.

[36] Hirano Y, Ozasa Y, Yamamoto T (2001). Hyperventilation and cold-pressor stress echocardiography for noninvasive diagnosis of coronary artery spasm. J Am Soc Echocardiogr.

[37] Pozzoli M, Traversi E, Cioffi G (1997). Loading manipulations improve the prognostic value of Doppler evaluation of mitral flow in patients with chronic heart failure. Circulation.

[38] Ishizu T, Seo Y, Kawano S (2008). Stratification of impaired relaxation filling patterns by passive leg lifting in patients with preserved left ventricular ejection fraction. Eur J Heart Fail.

[39] Yamada H, Oki T, Tabata T (1998). Differences in transmitral flow velocity pattern during increase in preload in patients with abnormal left ventricular relaxation. Cardiology.

[40] Yamada H, Kusunose K, Nishio S (2014). Pre-load stress echocardiography for predicting the prognosis in mild heart failure. JACC Cardiovasc Imaging.

[41] Lang RM, Badano LP, Mor-Avi V (2015). Recommendations for cardiac chamber quantification by echocardiography in adults: an update from the American Society of Echocardiography and the European Association of Cardiovascular Imaging. J Am Soc Echocardiogr..

[42] Brochet E, Detaint D, Fondard O (2011). Early hemodynamic changes versus peak values: what is more useful to predict occurrence of dyspnea during stress echocardiography in patients with asymptomatic mitral stenosis?. J Am Soc Echocardiogr.

[43] Magne J, Lancellotti P, Pierard LA (2010). Exercise-induced changes in degenerative mitral regurgitation. J Am Coll Cardiol.

[44] Lancellotti P, Magne J (2013). Stress echocardiography in regurgitant valve disease. Circ Cardiovasc Imaging.

[45] Magne J, Lancellotti P, Pierard LA (2010). Exercise pulmonary hypertension in asymptomatic degenerative mitral regurgitation. Circulation.

[46] Haluska BA, Short L, Marwick TH (2003). Relationship of ventricular longitudinal function to contractile reserve in patients with mitral regurgitation. Am Heart J.

[47] Magne J, Mahjoub H, Dulgheru R (2014). Left ventricular contractile reserve in asymptomatic primary mitral regurgitation. Eur Heart J.

[48] Kusunose K, Popovic ZB, Motoki H (2013). Prognostic significance of exercise-induced right ventricular dysfunction in asymptomatic degenerative mitral regurgitation. Circ Cardiovasc Imaging.

[49] Lancellotti P, Lebois F, Simon M (2005). Prognostic importance of quantitative exercise Doppler echocardiography in asymptomatic valvular aortic stenosis. Circulation.

[50] Lancellotti P, Lebois F, Simon M (2012). Determinants and prognostic significance of exercise pulmonary hypertension in asymptomatic severe aortic stenosis. Circulation.

[51] Bonow RO, Lakatos E, Maron BJ (1991). Serial long-term assessment of the natural history of asymptomatic patients with chronic aortic regurgitation and normal left ventricular systolic function. Circulation.

[52] Lee K, Daimon M, Kuwabara Y (2009). Prediction of the response to beta-blocker therapy in patients with dilated cardiomyopathy: comparison of 123I-MIBG scintigraphy and low-dose dobutamine stress echocardiography. J Echocardiogr.

[53] Scrutinio D, Napoli V, Passantino A (2000). Low-dose dobutamine responsiveness in idiopathic dilated cardiomyopathy: relation to exercise capacity and clinical outcome. Eur Heart J.

[54] Matsumoto K, Tanaka H, Kaneko A (2012). Contractile reserve assessed by three-dimensional global circumferential strain as a predictor of cardiovascular events in patients with idiopathic dilated cardiomyopathy. J Am Soc Echocardiogr.

[55] Lancellotti P, Lebrun F, Pierard LA (2003). Determinants of exercise-induced changes in mitral regurgitation in patients with coronary artery disease and left ventricular dysfunction. J Am Coll Cardiol.

[56] Lancellotti P, Magne J, Dulgheru R (2015). Clinical significance of exercise pulmonary hypertension in secondary mitral regurgitation. Am J Cardiol.

[57] Ypenburg C, Sieders A, Bleeker GB (2007). Myocardial contractile reserve predicts improvement in left ventricular function after cardiac resynchronization therapy. Am Heart J.

[58] Elliott PM, Anastasakis A, Borger MA (2014). 2014 ESC Guidelines on diagnosis and management of hypertrophic cardiomyopathy: the task force for the diagnosis and management of hypertrophic cardiomyopathy of the European Society of Cardiology (ESC). Eur Heart J.

[59] Peteiro J, Bouzas-Mosquera A, Fernandez X (2012). Prognostic value of exercise echocardiography in patients with hypertrophic cardiomyopathy. J Am Soc Echocardiogr.

[60] Yu EH, Omran AS, Wigle ED (2000). Mitral regurgitation in hypertrophic obstructive cardiomyopathy: relationship to obstruction and relief with myectomy. J Am Coll Cardiol.

[61] Nagueh SF, Smiseth OA, Appleton CP (2016). Recommendations for the evaluation of left ventricular diastolic function by echocardiography: an update from the American Society of Echocardiography and the European Association of Cardiovascular Imaging. J Am Soc Echocardiogr.

[62] Bossone E, D’Andrea A, D’Alto M (2013). Echocardiography in pulmonary arterial hypertension: from diagnosis to prognosis. J Am Soc Echocardiogr.

[63] Naeije R, Vanderpool R, Dhakal BP (2013). Exercise-induced pulmonary hypertension: physiological basis and methodological concerns. Am J Respir Crit Care Med.

[64] Argiento P, Chesler N, Mulè M, D’Alto M, Bossone E, Unger P, Naeije R (2010). Exercise stress echocardiography for the study of the pulmonary circulation. Eur Respir J.

[65] Kane GC, Hepinstall MJ, Kidd GM (2008). Safety of stress echocardiography supervised by registered nurses: results of a 2-year audit of 15,404 patients. J Am Soc Echocardiogr.

[66] Geleijnse ML, Krenning BJ, Nemes A (2010). Incidence, pathophysiology, and treatment of complications during dobutamine-atropine stress echocardiography. Circulation.

[67] Nishimura RA, Otto CM, Bonow RO (2014). 2014 AHA/ACC guideline for the management of patients with valvular heart disease: executive summary a report of the American College of Cardiology/American Heart Association Task Force on Practice Guidelines. Circulation.

[68] Pibarot P, Jean G, Dumesnil JG (2007). New concepts in valvular hemodynamics: implications for diagnosis and treatment of aortic stenosis. Can J Cardiol.

[69] Tribouilloy C, Lévy F, Rusinaru D (2009). Outcome after aortic valve replacement for low-flow/low-gradient aortic stenosis without contractile reserve on dobutamine stress echocardiography. J Am Coll Cardiol.

